# The influence of antibiotic prophylaxis on bacterial resistance in urinary tract infections in children with spina bifida

**DOI:** 10.1186/s12879-016-2166-y

**Published:** 2017-01-12

**Authors:** Sebastiaan Hermanus Johannes Zegers, Jeanne Dieleman, Tjomme van der Bruggen, Jan Kimpen, Catharine de Jong-de Vos van Steenwijk, Bas Zegers

**Affiliations:** 1 Máxima Medical Center, Post box 7777, 5500 MB Veldhoven, The Netherlands; 2 University Medical Center, Utrecht, The Netherlands; 3 Wilhelmina Children’s Hospital, University Medical Center, Utrecht, The Netherlands

## Abstract

**Background:**

Bacterial resistance to antibiotics is an increasingly threatening consequence of antimicrobial exposure for many decades now. In urinary tract infections (UTIs), antibiotic prophylaxis (AP) increases bacterial resistance. We studied the resistance patterns of positive urinary cultures in spina bifida children on clean intermittent catheterization, both continuing and stopping AP.

**Methods:**

In a cohort of 176 spina bifida patients, 88 continued and 88 stopped using AP. During 18 months, a fortnightly catheterized urine sample for bacterial pathogens was cultured. UTIs and significant bacteriuria (SBU) were defined as a positive culture with a single species of bacteria, respectively with and without clinical symptoms and leukocyturia. We compared the percentage of resistance to commonly used antibiotics in the isolated bacteria in both groups.

**Results:**

In a total of 4917 cultures, 713 (14.5%) had a positive monoculture, 54.3% of which were *Escherichia coli*. In the group stopping AP, the resistance percentage to antibiotics in UTI / SBU bacteria was lower than in the group remaining on AP, even when excluding the administered prophylaxis.

**Conclusion:**

Stopping antibiotic prophylaxis for urinary tract infections is associated with reduced bacterial resistance to antibiotics in children with spina bifida.

**Trial registration:**

ISRCTN ISRCTN56278131. Registered 20 December 2005.

## Background

Due to increasing antibiotic use, bacterial resistance has emerged as an significant healthcare problem. The use of broader antibiotics for infections is driven by local antibiotic susceptibility and is influenced by preventive measures, use of antibiotic prophylaxis and previous antibiotic use.

Prior to the recent AAP Guidelines, there has been a trend to prescribe antibiotic prophylaxis (AP) to prevent recurrence of urinary tract infections (UTIs) and possible subsequent renal parenchymal scarring in otherwise healthy children and children with congenital abnormalities of kidney and urological tract [1]. The time-delay in culture results leads to the prescription of broad-spectrum antibiotics in suspicion of a UTI. Bacterial resistance for an increasing number of antibiotics is therefore seen in UTIs [2–5].

In children with spina bifida, renal insufficiency due to urological impairments and recurrent UTIs has been the major cause of morbidity and mortality [6]. The introduction of clean intermittent bladder catheterization (CIC) in 1972 by Lapides et al. enabled more adequate bladder emptying and a significant decline in UTIs, renal insufficiency and the need for kidney transplantation [7]. However, since the introduction of CIC, many clinicians started prescribing AP to prevent CIC-related UTIs [8–10]. Due to the lack of general guidelines on the use of AP for children with spina bifida applying CIC [11], caretakers were guided by the clinical course in the individual patient to either continue or stop AP.

To study the value of AP in children with spina bifida applying CIC, we conducted the SPIN UTI trial in the Netherlands and Belgium [12]. In this trial 176 patients were randomized to continue or to stop AP. Stopping AP resulted in significantly more non-febrile UTIs (relative risk 1.44, 95% confidence interval 1.13 – 1.83, *p* 0.003). However, the absolute risk of UTI was low: on average, AP has to be administered for more than two years to prevent one non-febrile, and therefore non-renal scarring UTI. The recommendation from this study was to start AP upon diagnosing spina bifida, and to stop AP as soon as vesico-ureteral reflux is excluded, overactive bladder symptoms are treated with anticholinergics and CIC is properly implemented. Only spina bifida patients with persisting high grade vesico-ureteral reflux and severe overactive bladder despite anticholinergic medication, which results in significantly more UTIs, may benefit from continuation of AP [12].

During the SPIN UTI study, catheterized urine samples of the 176 patients were investigated fortnightly for a period of 18 months by dip stick and subsequent culture only if the dip stick was positive. This resulted in almost 5000 cultures. In case of a positive culture with one strain of bacteria (monoculture), antimicrobial susceptibility was determined. The main aim of the present analysis was to study the difference in antimicrobial susceptibility in positive urine cultures between patients continuing and stopping AP. Our hypothesis was that children stopping prophylaxis would have better susceptibility of bacteria to commonly used antibiotics.

## Methods

### Patients

All patients with a meningomyelocele (spina bifida) known at the outpatient clinics of Wilhelmina’s Children’s Hospital in Utrecht, the Netherlands and Gasthuisberg University Hospital in Leuven, Belgium were eligible for inclusion in the study, provided they had been on CIC and AP during the last 6 months. One hundred and seventy-six patients participated in the study. The study period was from February 2005 until March 2009. This study was approved and registered by the ISRCTN, trial number 56278131 (http://bit.ly/2hvP2Uq).

### Interventions

Patients were randomly allocated to continue or discontinue AP, using a computer based random concealed allocation scheme. Randomization was stratified for ages under and over 3 years, presence of vesico-ureteral reflux, gender and participating centre. Patients randomized to continuation of AP continued the individually prescribed type and dosage of antibiotics. The dosages and types were allowed to differ between patients according to antimicrobial susceptibility in pre-study cultures. Patients randomized to stopping of AP were instructed to discontinue the prescribed AP upon study start. The first urine sample was taken two weeks after stopping AP.

### Follow-up, outcome measurements, primary outcome definition

During an 18 months follow-up period, fortnightly dip sticks and urine cultures were performed after CIC by the patients themselves, their parents or their primary care takers.

The dip stick for both urinary leukocytes and nitrite (Combur2-LN®, Roche Diagnostics) was rated either as negative (no color change) or as positive (any color change) by the primary care takers. If the dipstick was positive for leukocytes and / or nitrite, a urine culture was performed using a Uricult™ test (Orion Diagnostica, Finland) with MacConkey and CLED media.

In Utrecht, the Netherlands, the Uricult™ was sent to the laboratory of clinical microbiology of the University Medical Centre Utrecht for a 24 hour incubation period at 37 °C (98°F). If rated positive, the Uricult was subcultured on a sheep blood agar for 72 hours followed by identification to the species level by automated bacterial identification and automated antimicrobial susceptibility testing providing MICs (Phoenix, Becton & Dickinson, MD). *Enterococcus* species were identified to the genus level by selective growth on bile esculin agar (BEA) and salt tolerance agar (STA). Enterococcal antimicrobial susceptibility testing was performed with disk diffusion. CLSI breakpoints for MICs and disk diffusion were used for interpretation. When negative, the Uricult™ was not subcultured.

In Leuven, Belgium, the 24 hour incubation period was performed at home by the primary care takers (parents or nurses), using a feeding bottle warmer (Philips®) at 37 °C. If rated positive, the Uricult™ was sent to the laboratory of clinical microbiology for the incubation and identification process. When rated negative at home, the Uricult™ was sent to the trained research nurse for professional review. When she rated the Uricult™ positive, it was yet sent to the laboratory for incubation.

Significant bacteriuria (SBU) was defined as more than 10,000 colony forming units of a single specimen per milliliter in the catheterized urine sample. Urinary tract infection (UTI) was defined as an SBU combined with a positive reading of leukocyturia on the dip stick and clinical symptoms, such as increasing incontinence, foul smell or cloudiness of the catheterized urine. Cultures presenting more than one species of bacteria, regardless of clinical symptoms, were considered as a contamination rather than SBU or UTI.

To avoid repeated calculations for bacterial resistance patterns on one period of persistent SBU, we considered multiple consecutive positive cultures (SBU) without clinical signs of UTI, and therefore no antibiotic treatment, as one ongoing colonization.

### Primary outcome

The primary outcome in this analysis was bacterial resistance of uropathogens in children with spina bifida on clean intermittent catheterization to commonly used antibiotics.

### Statistical methods

Main treatment effect analyses were published previously [12]. In brief, differences in rates of UTI between the two treatment groups were analyzed using Poisson regression and pointed at no clinically relevant difference in risk of SBU/UTI after stopping AP.

The present analysis represents a secondary analysis of bacterial resistance patterns observed in incubated cultures of urine samples of children with spina bifida and SBU or UTI and the influence of AP. Bacterial species, type of AP used and antibiotic susceptibility (both AP and non-AP antibiotic) were described according to the randomization group (intention-to-treat) and according to the actual use of antibiotics (per protocol).

Prevalence of AP and non-AP resistance in positive urine cultures was calculated as the number of cultures with resistant pathogens divided by the total number of positive cultures. In this calculation we assumed independence of multiple cultures within patients, which was deemed appropriate given the fact that we only included incident episodes of SBU/UTI. Results were stratified for actual AP use (yes/no) and type of AP used.

Differences in prevalence of pathogenic resistance between cultures with and without AP were statistically tested using the Generalized Linear Mixed Model (GLMM) module of SPSS which takes into account the repeated assessments in patients. We applied the binary logistic link function and a random effect for the individual intercept. As primary predictor of interest we included AP use (yes/no) at the time of SBU/UTI to explore the effect of AP use on the risk of resistance against non-AP antibiotics. In addition we explored the effect of country (the Netherlands vs. Belgium), SBU or UTI and gender as potential confounders by adding these variables to the model with AP. Results from this analysis were expressed as odds ratio (OR) with 95% confidence intervals (95% CI).

All analyses were performed using SPSS, version 19.0 and statistical significance was accepted at a two-sided *p*-value of 0.05.

## Results

Of the 176 participants, 88 were randomized to continue AP and 88 to stop using AP. In the latter group, 38 restarted AP during the 18 month study period, due to recurrent UTIs or specific parental request.

Not all patients complied with the fortnightly cultures in the entire study period of eighteen months. From a possible 6864 cultures if all 176 patients had complied with the protocol during the entire study period, 4917 urine samples were sent to and evaluated by the laboratories. Seven hundred and thirteen (14.5%) of these were positive single-strain cultures, of which 315 (44.2%) were considered a UTI due to clinical symptoms and leukocyturia on the dip stick. The remaining 398 (55.8%) were considered SBU, lacking clinical symptoms or leukocyturia (Table 1). No significant differences were seen between boys and girls.

**Table 1:** Frequency of positive cultures and percentages of uropathogens in 176 children with spina bifida on clean intermittent catheterization on and off antibiotic prophylaxis

	Total		Stop group		Continuing group		
	Number	%	Number	%	Number	%	*p*-value for stop versus continue
Patients with only negative urine tests	23	13.1	11	12.5	12	13.6	
Patients with one or more positive cultures	153	86.9	77	87.5	76	86.4	Pearson chi-square 0.635
SBU	137	77.8	70	79.5	67	76.1	1,000
UTI	107	60.8	55	62.5	52	59.1	0.643
Number of positive cultures	713	14.5	370		343		
SBU	398	55.8	199	53.8	199	58.0	
UTI	315	44.2	171	46.2	144	42.0	
		CI		CI		CI	
Mean number of positive cultures per patient (95% CI)	4.66	(4.1-5.2)	4.81	(4.1-5.5)	4.51	(3.8-5.2)	negative binominal analysis 0.573
SBU	2.60	(2.3-2.9)	2.58	(2.2-3.0)	2.62	(2.1-3.1)	0.915
UTI	2.06	(1.7-2.4)	2.22	(1.7-2.7)	1.89	(1.4-2.4)	0.364
Uropathogens in positive cultures (% of positive cultures)		%		%		%	
E.coli	387	54.3	200	54.1	187	54.5	Pearson chi-square 0.429
Non E.coli	326	45.7	170	45.9	156	45.5	
*Gram negative*	631	88.5	326	88.1	305	88.9	0.901
Enterobacteriaceae							
AmpC negative							
*E.coli*	387	54.3	200	54.1	187	54.5	
*Klebsiella pneumoniae*	42	5.9	21	5.7	21	6.1	
*Proteus mirabilis*	34	4.8	15	4.1	19	5.5	
*Klebsiella oxytoca*	21	2.9	16	4.3	5	1.5	
*AmpC positive*							
*Enterobacter cloacae*	18	2.5	10	2.7	8	2.3	
*Citrobacter freundii*	17	2.4	11	3.0	6	1.7	
Non fermenting bacilli							
*Pseudomonas auroginosa*	47	6.6	29	7.8	18	5.2	
Other	65	9.1	24	6.5	41	12.0	
*Gram positive*	82	11.5	44	11.9	38	11.1	0.762
Enterococcal species	56	7.9	30	8.1	26	7.6	
*Staphylococcus aureus*	18	2.5	10	2.7	8	2.3	
Other gram positive	8	1.1	4	1.1	4	1.2	
CI = 95% confidence interval							
SBU = significant bacteriuria							
UTI = urinary tract infection							

The most common pathogen seen in about half of both SBU and UTI was *Escherichia coli (E.coli)* (54.3%). The other 45.7% consisted of other well-known uropathogens, like *Klebsiella* species (8.8%), *Enterococcus* species (7.9%), *Pseudomonas aeruginosa* (6.6%), *Proteus mirabilis* (4.8%) and 17.6% of less common pathogens (Table 1). Of the 713 single strain cultures only 82 (11.5%) were Gram-positive bacteria, mostly *Enterococcus* species and *Staphylococcus aureus*. Again, there were no differences between boys and girls in pathogens in either group.

Almost half of the cultures were performed in patients randomized to continue AP (*n* = 343, 48.1%), the remaining 370 cultures (51.9%) were from patients randomized to stopping AP. However, 79 (21%) cultures in the stop group were performed after AP was restarted due to recurrent UTIs or specific parental request. Thus, the majority of SBU and UTI occurred while using AP (*N* = 422, 59.2%), mostly trimethoprim and/or nitrofurantoin (Table 2).

**Table 2:** Percentages of resistance of gram-negative uropathogens against commonly used antibiotics per administered antibiotic prophylaxis

	No prophylaxis	Nitrofurantoin prophylaxis	Trimetoprim prophylaxis	Nitrofurantoin and trimetoprim prophylaxis	Ciprofloxacin prophylaxis
Number of gram-negative cultures	228	166	131	84	15
Antibiotic tested	% resistant				
Amoxicillin	57.0	57.3	71.3*	79.0**	55.6
Amoxicillin/clavulanic acid	23.2	21.5	26.7	40.9**	46.2
Piperacillin	56.6	38.6*	62.5	70.4	41.7
Piperacillin/tazobactam	5.6	4.9	3.4	23.3***	8.3
Cefazolin	17.3	14.0	14.9	29.0	66.7**
Cefuroxim	9.0	21.5**	5.2	14.3	70.0***
Ceftazidim	3.4	12.9*	1.7	5.5	9.1
Ceftriaxon	1.4	7.9*	0.0	3.1	10.0
Meropenem	0.0	2.4	1.6	1.2	8.3
Amikacin	0.6	9.5**	0.8	2.5	0.0
Gentamicin	3.2	4.4	2.6	6.3	0.0
Tobramycin	3.1	6.0	1.6	8.6*	0.0
Ciprofloxacin	3.8	7.2	12.0	2.5	58.3***
Norfloxacin	5.1	8.9	11.6	3.8	63.6***
Levofloxacin	5.9	10.2	11.8	2.5	53.8***
Trimetoprim	38.5	38.7	90.5***	83.3***	55.6
Co-trimoxazol	32.1	30.9	79.1***	71.8***	38.5
Nitrofurantoin	13.2	56.1***	11.6	14.3	77.8***

Legend: GLMM used to compare risk of resistance relative to no prophylaxis used, * *p* < 0.05, ** *p* < 0.01, *** *p* < 0.001

### Microbial resistance

Microbial resistance against any antibiotic was present in 65.2% of SBU/UTIs, and significantly more prevalent in urine cultures taken in children with spina bifida on AP (72.2%) than in children without AP (53.3%).

In Table 3, determination of resistance patterns to commonly used antibiotics performed on positive urinary cultures with Gram negative bacterial species, tested more than ten times in our patient group, is shown. There were too few Gram positive urinary cultures results to significantly differentiate resistance percentages between the groups on and off AP. The main result shown in this table is that use of AP increases the risk of resistance compared to stopping AP: the percentages of resistance to a specific antibiotic in any bacteria found in the urine cultures were higher when using AP. GLMM analysis estimated the risk of resistance against one or more antibiotics (including the AP) to be 2.3 (95% CI 1.6-3.1) fold higher while using AP relative to not using AP. Adding country, gender or type of culture (SBU or UTI) in the GLMM analysis did not change this estimate. Excluding resistance to the administered AP changed the estimate slightly to OR 1.7 (95% CI 1.2-2.3) for AP use relative to no AP use.

**Table 3:** Percentages of resistance against commonly used antibiotics in positive gram-negative urine cultures on and off antibiotic prophylaxis

		Gram negative uropathogens							
		AmpC negative				AmpC positive		Non-fermenting	
		*E.coli*	*Klebsiella pneumoniae*	*Proteus mirabilis*	*Klebsiella oxytoca*	*Enterobacter cloacae*	*Citrobacter freundii*	*Pseudomonas aeroginosa*	*p*-value AP+ vs AP-
	Number of cultures	387	42	34	21	18	17	47	
Antibiogram									
*Penicillines*									
Amoxicillin	total	66.7	NC	24.2	NC	NC	NC	NC	0.031
	AP+	73.8	NC	11.5	NC	NC	NC	NC	
	AP-	56.3	NC	#	NC	NC	NC	NC	
Amoxicillin/clavulanic acid	total	29.7	9.5	6.1	9.5	NC	NC	NC	0.172
	AP+	32.8	9.1	7.7	#	NC	NC	NC	
	AP-	25.3	#	#	14.3	NC	NC	NC	
Piperacillin	total	68.2	NC	9.1	NC	NC	NC	2.6	0.950
	AP+	73.5	NC	9.5	NC	NC	NC	3.1	
	AP-	59.5	NC	#	NC	NC	NC	#	
Piperacillin/tazobactam	total	7.8	17.1	3.0	5.3	#	#	2.2	0.176
	AP+	10.3	15.6	3.8	#	#	#	2.7	
	AP-	4.0	#	#	7.7	#	#	#	
*Cephalosporines*									
Cefazolin (1)	total	17.9	43.8	4.5	56.3	NC	NC	NC	0.346
	AP+	20.6	54.5	0.0	#	NC	NC	NC	
	AP-	13.5	#	#	50.0	NC	NC	NC	
Cefuroxim (2)	total	11.4	45.2	3.0	14.3	NC	NC	NC	0.016
	AP+	13.5	51.5	0.0	#	NC	NC	NC	
	AP-	8.2	#	#	9.1	NC	NC	NC	
Ceftazidim (3)	total	1.7	21.7	4.3	5.9	NC	NC	4.3	0.161
	AP+	2.6	29.4	4.5	#	NC	NC	2.6	
	AP-	0.0	#	#	4.5	NC	NC	#	
Ceftriaxon (3)	total	1.9	19.4	0.0	5.9	#	NC	#	0.148
	AP+	2.5	25.0	0.0	#	#	NC	#	
	AP-	0.8	#	#	9.1	#	NC	#	
*Other bactolactam*									
Meropenem	total	0.0	0.0	0.0	0.0	0.0	0.0	7.5	0.461
	AP+	0.0	0.0	0.0	#	#	#	9.1	
	AP-	0.0	#	#	0.0	#	?	#	
*Aminoglycosides*									
Amikacin	total	1.9	19.0	4.2	0.0	0.0	0.0	2.5	0.054
	AP+	2.6	26.7	4.3	#	#	#	3.0	
	AP-	0.9	#	#	0.0	#	?	#	
Gentamicin	total	4.7	2.4	0.0	0.0	0.0	0.0	0.0	0.500
	AP+	4.4	3.0	0.0	#	#	#	0.0	
	AP-	4.8	#	#	0.0	#	0.0	#	
Tobramicin	total	4.9	4.8	3.0	0.0	0.0	0.0	2.1	0.194
	AP+	4.8	6.1	3.8	#	#	#	2.6	
	AP-	4.4	#	#	0.0	#	0.0	#	
*Fluorquinolones*									
Ciprofloxacin	total	7.8	52.9	0.0	0.0	0.0	0.0	5.0	0.021
	AP+	10.2	63.6	0.0	#	#	#	6.1	
	AP-	3.6	#	#	0.0	#	0.0	#	
Norfloxacin	total	7.7	52.9	0.0	0.0	0.0	0.0	9.4	0.047
	AP+	9.7	63.6	0.0	#	#	#	11.1	
	AP-	4.5	#	#	0.0	#	0.0	#	
Levofloxacin	total	9.4	29.3	0.0	0.0	0.0	0.0	9.4	0.057
	AP+	10.6	33.3	0.0	#	#	#	11.1	
	AP-	7.7	#	#	0.0	#	0.0	#	
*Other antibiotics*									
Trimetoprim	total	66.6	88.2	34.8	23.5	16.7	66.7	NC	<0.001
	AP+	80.7	63.6	36.4	#	#	#	NC	
	AP-	42.9	#	#	18.2	#	90.9	NC	
Cotrimoxazol	total	55.7	52.4	33.3	19.0	11.8	47.1	NC	<0.001
	AP+	69.7	51.5	30.8	#	#	#	NC	
	AP-	35.4	#	#	14.3	#	10.1	NC	
Nitrofurantoin	total	16.4	90.5	NC	28.6	64.7	0.0	NC	<0.001
	AP+	22.4	93.9	NC	#	#	#	NC	
	AP-	7.7	#	NC	21.4	#	0.0	NC	

Legend: # = not calculated as less than ten samples tested, NC = Not considered due to intrinsic resistance or uncommon clinical drug/bug combination

Table 2 depicts the association between type of AP on resistance patterns in 624 Gram negative cultures. Due to statistical insignificance, we left out the few Gram-negative cultures on other AP than trimethoprim, nitrofurantoin, ciprofloxacin or a combination of trimethoprim and nitrofurantoin.

## Antibiogram of uropathogens and the influence of antibiotic prophylaxis on resistance

### Penicillins

In our study cohort, resistance against amoxicillin and piperacillin was common with a overall prevalence of 66.7% and 68.2% in *E.coli* UTIs (Table 3). Stopping AP decreased the percentage of resistance in *E.*coli UTIs against amoxicillin and piperacillin from respectively 73.8 and 73.5% to 56.3% and 59.5%. Resistance against amoxicillin/clavulanic acid (29.7%) and piperacillin/tazobactam (7.8%) was less common in *E. coli* UTIs, as well as in other Gram negative UTIs. When discontinuing AP, the prevalence of resistance against amoxicillin/clavulanic acid and piperacillin / tazobactam decreased to respectively 22.7 and 5.5% (Table 2).

### Cephalosporins

Gram negative bacteria such as *E.coli* showed moderate resistance for first and second generation cephalosporins (17.9% and 11.4% respectively) in our cohort of spina bifida patients, whereas for third generation cephalosporins *E.coli* had significantly lower resistance of 1.7-1.9% (Table 3). In UTIs with the uropathogen *Klebsiella pneumoniae* however, one in five is resistant to a third generation cephalosporin. Compared to not using AP, trimethoprim (0% and 5%) and nitrofurantoin alternating with trimethoprim (3% and 15%) as AP did not significantly influence resistance to second and third generation cephalosporins. However, the resistance for second generation cephalosporins increased significantly when using nitrofurantoin (21%) or ciprofloxacin (70%) as AP (Table 2).

### Fluoroquinolones

In our cohort there was an overall low resistance of around 5% for fluoroquinolones in Gram negative bacteria while not using AP (Table 2). This was negatively influenced by prophylaxis: nitrofurantoin and trimethoprim prophylaxis doubled the resistance percentage (7.1 and 13.0% for ciprofloxacin respectively) (Table 2). When trimethoprim and nitrofurantoin were taken alternately, the resistance rate remained as low as without AP. The use of ciprofloxacin as AP was associated with a sharp increase in fluoroquinolones resistance (58.3-63.6%) (Table 2).

### Trimethoprim/sulfamethoxazole

Even without AP, in our cohort *E.coli* bacteria had high resistance for both trimethoprim (42.9%) and trimethoprim/sulfamethoxazole (35.4%) (Table 3). Resistance obviously increased when using AP involved trimethoprim (90.1% and 77.7% respectively) (Table 2). Nitrofurantoin as AP however was not associated with increased resistance for trimethoprim (38.1%) or trimethoprim/sulfamethoxazole (30.7%) in Gram negative bacteria, whereas ciprofloxacin as AP only mildly increased resistance to trimethoprim (45.5%) and trimethoprim/sulfamethoxazole (33.3%) (Table 2).

### Aminoglycosides

There was a low resistance rate for intravenous aminoglycosides in our cohort (3%), not influenced by AP nitrofurantoin (4%) or trimethoprim (3%). When using ciprofloxacin as AP, the Gram negative bacteria also remained sensitive to gentamicin, amikacin or tobramycin (Table 3).

### Nitrofurantoin

Without AP, 13.2% of UTIs were resistant for nitrofurantoin treatment (Table 2). This resistance for nitrofurantoin treatment remained stable when using trimethoprim as AP (11.6%), while resistance significantly increased when using ciprofloxacin (77.8%) or nitrofurantoin itself (56.1%) as AP (Table 2).

The presence of resistance was not associated with age or gender. Microbial resistance against the prophylactic AP was not 100%: bacterial pathogens were still sensitive for treatment with the used AP in 43.9% of nitrofurantoin, 41.7% of ciprofloxacin and 9.5% of trimethoprim prescribed patients (Table 2).

## Discussion

Bacterial resistance is an emerging and hazardous phenomenon occurring with ever-increasing use of antibiotics. Antibiotic prophylaxis (AP) administered to prevent recurrent urinary tract infections (UTIs) contributes to this resistance [13], although it has been proven that AP does not decrease the risk of renal scarring [1].

We compared bacterial susceptibility patterns in positive urine cultures in children with spina bifida and CIC continuing or stopping AP. Overall, our study showed a decrease in resistance to commonly used antibiotics when AP is stopped, confirming our hypothesis. Even when the administered AP is excluded from these calculations, the number of antibiotics to which the cultured pathogen is resistant remains higher in the continuing group. These findings in spina bifida patients on CIC is in accordance with previous studies for resistance patterns comparing AP to no AP in patients with community-acquired UTIs [14–18]. The fact that a particular class of antibiotics is associated with resistance towards other classes of antibiotics might be explained by the observation that bacterial resistance traits can be linked [19, 20].

*E.coli* accounts for 75-90% of community-acquired UTIs [21, 22], whereas *E.coli* is responsible for only 54.3% of the SBUs and UTIs in our specific population of children with spina bifida, with higher percentages of other uropathogens causing SBU/UTI. This difference is a common feature in non community-acquired UTIs, as described in previous studies in non-spina bifida patients, from Landhani et al (40% *E.coli* in children with underlying pathology), Lutter et al (58% *E.coli* in non-spina bifida children on AP) and Wagenlehner et al (35-60% *E.coli* in adult hospital-acquired UTIs due to catheterization with introduction of alternative pathogens) [15, 16, 23].

### Choice of antibiotic prophylaxis in children with spina bifida

Our SPIN UTI study has shown that, whenever safe according to urological care, AP to prevent UTIs should be stopped in children with spina bifida. In a previous article we have shown that every child has to take two years of daily AP to prevent one extra non-febrile, non-scarring UTI [12], and this current study reveals a significant improvement in susceptibility to any necessary antibiotic treatment for a UTI when stopping AP. This article therefore emphasizes the necessity to stop the use of AP in children whenever possible to prevent bacterial resistance, especially since AP has proven not to prevent renal scarring [1].

When however, for reasons of recurrent UTIs, a persistent overactive bladder or high grade vesico-ureteral reflux, AP is a necessity, the choice of prophylaxis has impact on the bacterial resistance to commonly used therapeutic antibiotics. Trimethoprim as AP has the least negative influence on bacterial resistance: in our study cohort, the susceptibility of most therapeutic antibiotics remains relatively stable, except for fluoroquinolones, trimethoprim itself and trimethoprim/sulfamethoxazole. In our study, the use of nitrofurantoin as AP is associated with an increased resistance to cephalosporines, aminoglycosides and fluoroquinolones, with an increased risk of treatment failure, compared to non-AP patients. Particularly AP with fluoroquinolones is associated with a high percentage of resistance, especially to therapeutic oral antibiotic possibilities when necessary, and should therefore be discouraged.

### Choice of therapeutic antibiotics in children with spina bifida

First consideration in choosing an appropriate antibiotic when a UTI is suspected or confirmed is the manner of administration: when clinically not ill, oral antibiotic treatment is adequate, whilst in sick children with spina bifida due to a UTI intravenous administration of antibiotics is often necessary. This determines the choice of antibiotic treatment, along with previous culture results and resistance patterns, presence or absence of fever and recently prescribed AP. In our study cohort, nitrofurantoin is first choice medication for a UTI without fever or recent AP. Without fever but with prophylaxis, in children with trimethoprim as AP nitrofurantoin is still first choice. In other AP and in children with fever on or off AP, oral treatment for UTI depends on local susceptibility, with ciprofloxacin and cefuroxim as antibiotics with high a priori chance of treatment success in our study cohort. When intravenous treatment is warranted, a third generation cephalosporin, fluoroquinolon or carbapenem is possible. However, we emphasize that the choice of therapeutic antibiotics depends on local susceptibility and individual resistance patterns in previous urinary cultures.

The strength of this study is the large number of adequate catheterized urinary cultures in a cohort of susceptible children with spina bifida. Remarkable is the relatively high percentage of susceptibility of bacteria for the already administered AP.

## Conclusion

Discontinuation of antibiotic prophylaxis decreases bacterial resistance for commonly used antibiotics in children with spina bifida on clean intermittent catheterization should be pursued to prevent bacterial resistance, long term side effects of prophylactic antibiotics and the need for hospital admissions for broad spectrum intravenous antibiotics.

## Abbreviations

AP: Antibiotic prophylaxis
CI: Confidence interval
CIC: Clean intermittent catheterization
GLMM: Generalized Linear Mixed Model
OR: Odds ratio
SBU: Significant bacteriuria
UTI: Urinary tract infection
