# Erratum to: The influence of antibiotic prophylaxis on bacterial resistance in urinary tract infections in children with spina bifida

**DOI:** 10.1186/s12879-017-2268-1

**Published:** 2017-02-27

**Authors:** Sebastiaan Hermanus Johannes Zegers, Jeanne Dieleman, Tjomme van der Bruggen, Jan Kimpen, Catharine de Jong-de Vos van Steenwijk

**Affiliations:** 1 Máxima Medical Center, Post box 77775500, MB Veldhoven, The Netherlands; 2 University Medical Center, Utrecht, The Netherlands; 3 Wilhelmina Children’s Hospital, University Medical Center, Utrecht, The Netherlands

## Erratum

In this version of this article that was originally published [1] there was an error in the author list with an alternative name for the first author, ‘Sebastiaan Hermanus Johannes Zegers’, being incorrectly listed as the last author, ‘Bas Zegers’. The original article has been updated to delete the duplicate author name, ‘Bas Zegers’, and to correctly list ‘Sebastiaan Hermanus Johannes Zegers’ as the corresponding author.
